# Cadaveric biomechanical testing of torque - to - failure magnitude of Bilateral Apical Vertebral Derotation maneuver in the thoracic spine

**DOI:** 10.1371/journal.pone.0221494

**Published:** 2019-08-26

**Authors:** Rafal Pankowski, Marek Roclawski, Marcin Ceynowa, Tomasz Mazurek, Lechoslaw Ciupik, Agnieszka Kierzkowska

**Affiliations:** 1 Department of Orthopaedics, Medical University of Gdansk, Gdansk, Poland; 2 LfC/IBeMT—LfC Medical/IBeMT Institute of Bioengineering and Medical Technologies, Zielona Gora, Poland; University of California Davis, UNITED STATES

## Abstract

It remains unclear what is the real safe limit of torque magnitude during Bilateral Apical Vertebral Derotation (BAVD) in thoracic curve correction. Up to author’s knowledge there is no study except this one, to reproduce in–vivo real measurements and intraoperative conditions during BAVD maneuver. The objective of this study was to evaluate the torsional strength of the instrumented thoracic spine under axial rotation moment as well as to define safety limits under BAVD corrective maneuver in scoliosis surgery. 10 fresh, full-length, young and intact human cadavers were tested. After proper assembly of the apparatus, the torque was applied through its apical part, simulating thoracic curve derotation. During each experiment the torque magnitude and angular range of derotation were evaluated. For more accurate analysis after every experiment the examined section of the spine was resected from the cadaver and evaluated morphologically and with a CT scan. The average torque to failure during BAVD simulation was 73,3 ± 5,49Nm. The average angle of BAVD to failure was 44,5 ± 8,16°. The majority of failures were in apical area. There was no significant difference between the fracture occurrence of left or right side of lateral wall of the pedicle. There was no spinal canal breach and/or medial wall failure in any specimen. The safety limits of thoracic spine and efficacy of BAVD for axial plane correction in the treatment of Adolescent Idiopathic Scoliosis (AIS) were established. It provided qualitative and quantitative information essential for the spinal derotation under safe loading limits.

## Introduction

Derotation of the spine maneuver has recently gained popularity as it provides efficient control of the spine in the transverse plane creating better 3 –dimensional correction of the curve [1–3]. Also, it seems to be the most challenging and still not very well-known part of surgical correction in Adolescent Idiopathic Scoliosis (AIS), therefore technical development has progressed to improve this maneuver. A Direct Vertebral Rotation (DVR) system introduced by Suk in 2004, applies direct rotational force on Apical Vertebrae (AV) in the opposite direction to the rod rotation maneuver and to that of the deformity. It has proven good corrective abilities in axial plane as well as an effective rib hump reduction [1]. Many of DVR systems have been widely utilized for the surgical treatment of patients with AIS [2–6]. Recently a Bilateral Apical Vertebral Derotation (BAVD) introduced by Chang and Lenke [6] came into practice. It consists of manipulating both concave apical screws and convex periapical screws as a “quadrilateral frame” to bilaterally simultaneously manipulate the spine, thereby effectively correcting scoliotic deformity. Nowadays, a Computed Tomography imaging (CT) is the only objective evaluation of spinal rotation, however even with this method a precise measurement is difficult [7–10]. With the use of Intraoperative Computed Tomography (ICT) it was shown that derotational capability of DVR devices is not only relatively slight (11,5% to 33%) but also dependent from the implant density [11,12]. It was also shown that connecting of additional pedicle screws has a near linear effect on the force that can be applied to derotate a segment of the spine [13]. In larger or stiff scoliotic curves, an immense derotational and translational forces have to be applicated to apical and juxtaapical vertebrae in order to achieve significant BAVD [14]. Last but not least is that the concave rod tends to create the new technical axis of rotation, which is blocked by the natural axis of rotation of the spine that passes through the center of rotation of every vertebra and intervertebral disc [1,11,12,15,16]. For the effective DVR or BAVD maneuvers, a device with large lever arms has to be attached to the pedicle screws at multiple levels of the spine through which surgeon can apply significant torsional loads to the curve. The safety of these procedures has not been established yet and has been questioned due to possible pedicle fractures or other serious intraoperative complications such as deterioration of neural structures in the spinal canal or aortic abutment due to plowing of pedicle screws [17–19]. For the obvious reason intraoperative biomechanical testing of torsional failure strength on scoliotic spine are not to be performed. Although in-vitro biomechanical studies of the spine specimen’s response for torsional forces such as during DVR maneuver are accepted as reasonable for making in-vivo predictions, it is understood that spinal motions and responses may only be estimates of true in-vivo measurements with limited clinical importance [13,20,21]. There are studies concerning biomechanics of scoliosis that are based on mathematical model, estimating only the failure torques with lack of the precise data [22–24]. However, to date there is still lack of safe limits of the magnitude of torque of DVR or BAVD so they need to be established through the best possible quality intraoperative simulation of cadaver spine that will sufficiently quantify the failure loads (BAVD to failure). Up to author’s knowledge despite the large variations amongst the biomechanical studies of derotation of the spine, there is no study except this one, where fresh, young and intact cadavers were used for BAVD critical torque testing. This helped us to reproduce in–vivo real measurements and intraoperative conditions. In this original study a novel spinal custom made derotation tool and testing methods were introduced. These challenged the previous biomechanical studies and improved upon their limitations. Moreover, what is even more important, the clinical applicability of this preclinical biomechanical study could lead to the invention of Apical Derotator, which would be used routinely during scoliosis surgery in the clinical field.

## The aim

The purpose of this study was to examine biomechanical differences in transverse plane of thoracic pedicle screws in both medial and lateral directions as well as to evaluate the torsional strength of the instrumented thoracic spine under axial rotation moment and to define safety limits under typical derotation maneuvers in scoliosis surgery.

## Methods

**Fig 1:**
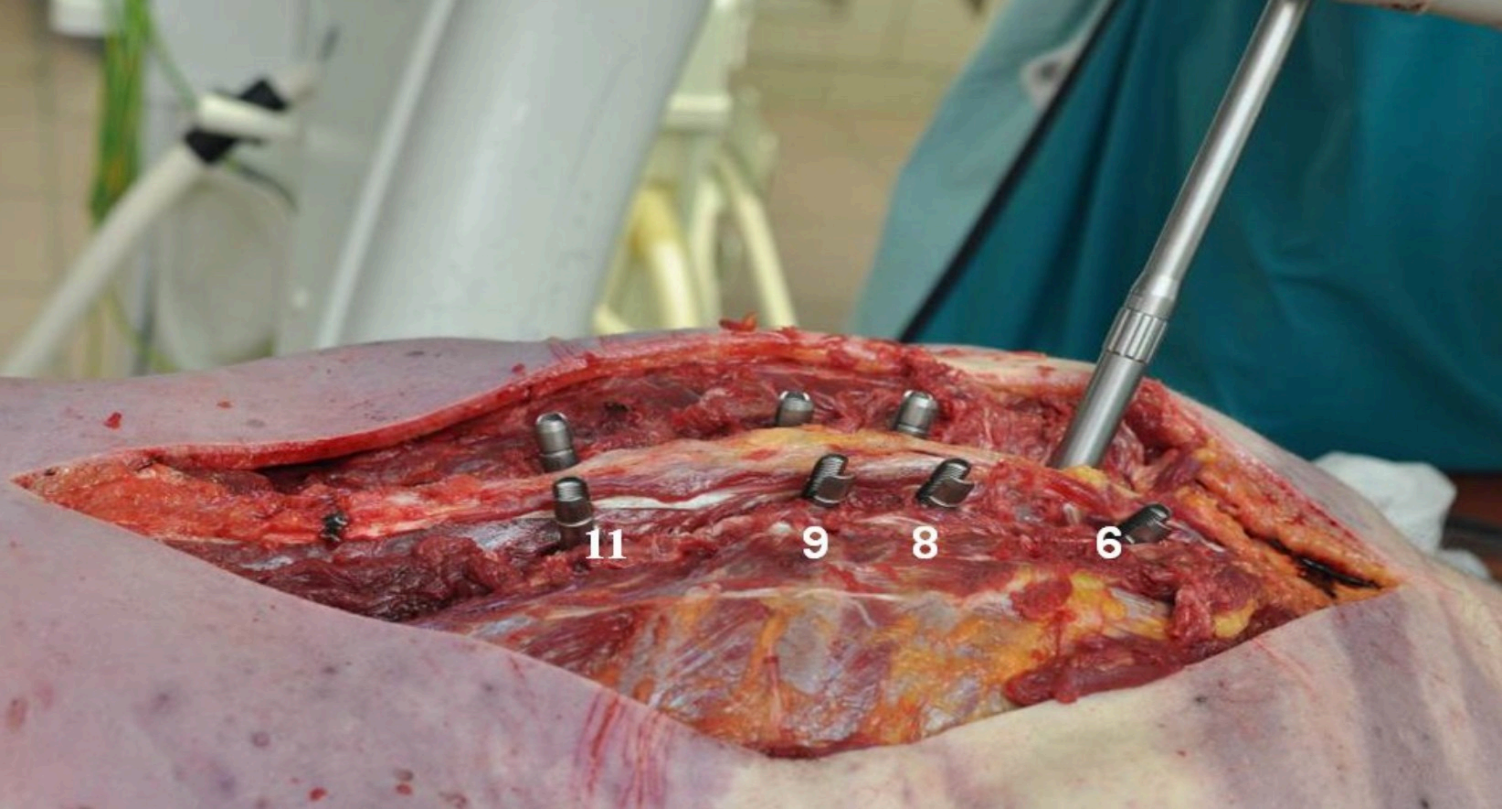
Pedicle screws placed in Th6, 8, 9, 11.

Institutional review board approval for the study (NKBBN/242/2013) was obtained locally from the Medical University of Gdansk Ethics Committee. 10 fresh, full-length human cadavers were obtained from the Forensic Medicine Department of the Medical University of Gdansk (DFM of MUG). The average age while death was 34,6ys (21–40). There were one female and nine male specimens. For the obvious reason, fresh human cadavers with scoliotic deformity were not available. Every specimen was intact and had maintained the integrity of all: muscles, posterior elements, bony structures, intervertebral discs, stabilizing ligaments, ribs, chest wall and whole trunk. In every case by order of the district prosecutor's offices they have already been made medico-legal autopsies at the DFM of MUG. The causes of death were by an overdose of drugs, alcohol, suicide, suffocation or hanging. Biomechanical tests were carried out after 48–72 hours after sections. With specimens lying prone a standard posterior midline approach was performed over the levels to be instrumented (Th6 to Th11). Then a meticulous exposure of the posterior elements was performed out to the tips of the transverse processes. After decortication of the posterior structures the proper placement of all bilateral, mono-axial pedicle screws was performed in every case at the same levels (Th6,8,9,11) that reflect the most common type of the AIS curve (Lenke I–right thoracic single curve scoliosis) (Fig 1).

**Fig 2:**
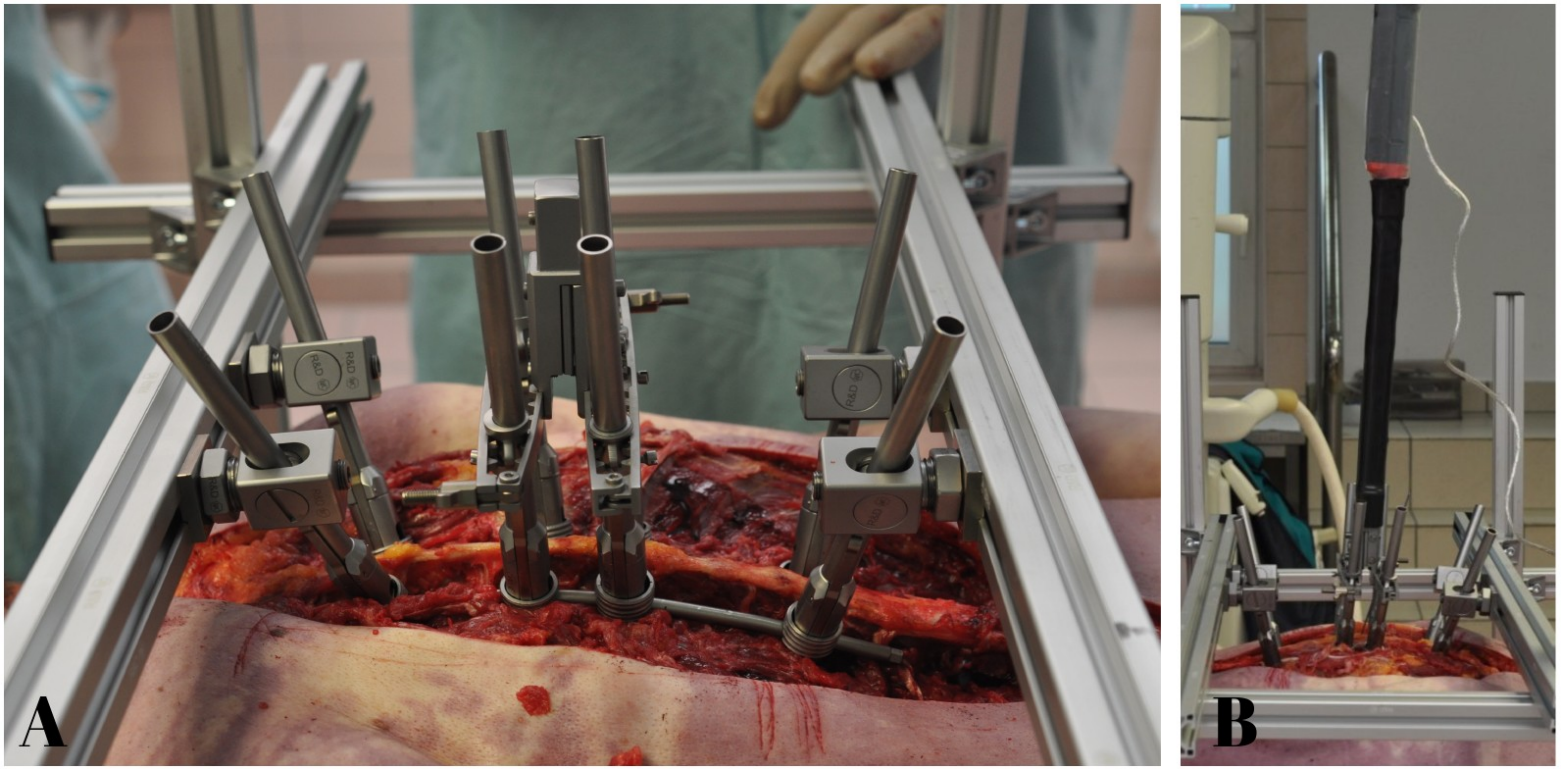
Custom made simulated BAVD experimental setup. **A)** Stabilizing frame with connectors for the upper and lowermost screws, 8 tubular implant holders that attach to the tulip of each pedicle screw, a buckle that connects the tubular implant holders together, and derotator bridge handle, screw/rod/tubular implant holder system SGL (LfC, Zielona Gora, Poland) **B)** Dynamometric torque wrench (BDS 30-300Nm ± 0,25, ISO 6789.2003, EN ISO 6789/2003) assembled to the derotator bridge handle connected to a computer dedicated to the collection of the strain signals.

All pedicle screws were inserted with fluoroscopic—guided placement technique that we routinely use during scoliosis surgery. After identification of starting points at particular levels a specially designed custom-made sharp tip pins were used for both: to create a posterior cortical breach and to probe down the pedicle into the vertebral body. Using the fluoroscopic guidance (C-arm) at antero-posterior view parallel to the superior endplate a proper entry site was located from the lateral side of the pedicle shadow. After entry site hole perforation with the use of anatomic landmarks, the pin was introduced medially and downward using intermittent antero-posterior views until it approached to the medial wall of the pedicle shadow. This was to prevent medial canal encroachment through the ventral lamina. Than a C-arm lateral image was taken to control the depth of the pin and when it proceed to the vertebral body (usually 20 to 25 mm) a pin was removed and a ball-tipped pedicle sounder was utilized to palpate 5 distinct bony borders: the floor and 4 walls in a typical and well described fashion. With intraosseous borders confirmed, pedicle screw length was assessed, and the pedicle was under—tapped with a 5-mm diameter tap. Once again, the pedicle sounder was used after tapping to confirm an intraosseous position. All cadaver’s pedicle sizes allowed to use the same diameter pedicle screws. We used in every experiment 8 pedicle screws SGL (LfC, Zielona Gora, Poland) of 6mm of diameter with appropriate individually measured lengths. Given our predominance of male specimens, these accepted larger screws than routinely used in the apex of the concavity of scoliotic spines. All our screws were then inserted in the same alignment (Th6,8,9,11) being careful to not to breach the anterior cortex. After the screw placement, 6 mm titanium rod was applied always on the left (concave) side of the specimen and tightened with nuts at both ends of the instrumentation except the apical screws (Th8,9) so they should be freely gliding in the rod there. A special custom-made vertebral derotation simulator was used during this study, designed and fabricated by (LfC, Zielona Gora, Poland). The simulator was designed to measure a maximum torque of derotation during BAVD maneuver simulation. The components of this system were consisted of: a stabilizing frame with connectors for the upper and lowermost screws, 8 tubular implant holders that attach to the tulip of each pedicle screw, a buckle that links the tubular implant holders together, and a derotator bridge handle that can be joined together with derotator torque gauge. After screws and rod placement, a stabilizing frame was assembled and fixed to the table at both sides of a specimen. Then tubular implant holders were attached one to each screw. Then the uppermost screws/implant holder’s assembly (in Th6) and lowermost (in Th11) were fixed to the stabilizing frame by special connectors creating a stiff frame. The apical screws/tubular implant holder’s system (Th8 and Th9) were connected together with special interlink buckles creating a quadrangular linkage with the pedicle screws and a tightened quadrilateral frame for “en block” BAVD maneuver simulation (Fig 2A).

**Fig 3:**
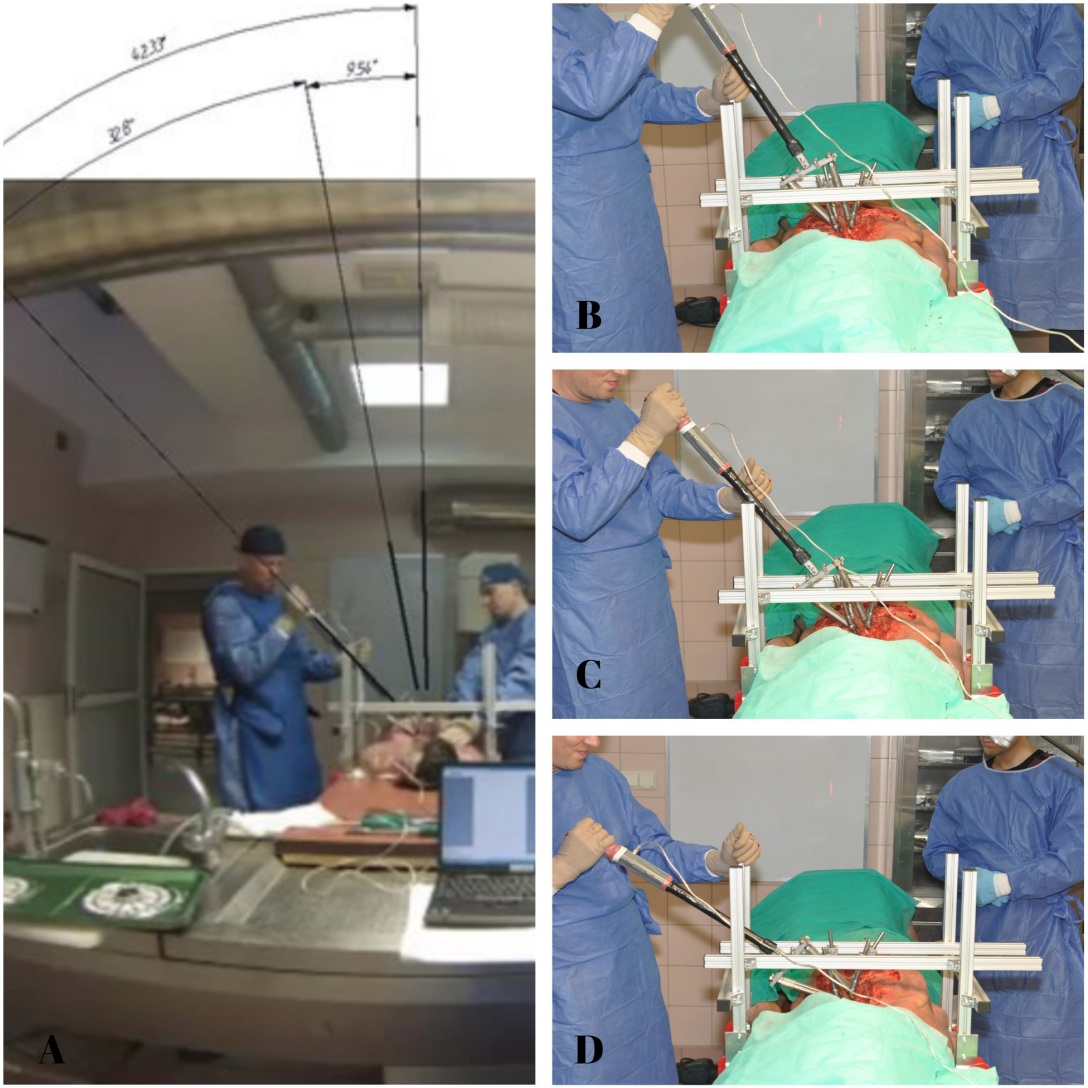
BAVD to failure testing. **A)** Camera view of the range of angle of simulated BAVD **B,C,D)** Intraoperative simulation of BAVD—to—failure after the end of the experiment with constant increase in the angle of BAVD simulation until failure.

Before the beginning of measurement of the simulated maximal torque loads a dynamometric torque wrench (BDS 30-300Nm ± 0,25, ISO 6789.2003, EN ISO 6789/2003) was assembled to the derotator bridge handle and connected to computer specially dedicated to the collection of strain signals (Fig 2B). The torque was then applied through the apical part of the apparatus, in anti-clockwise direction from the left to the right side (simulating AIS right thoracic curve derotation) effectively sharing the load across two apical vertebral bodies. This allowed to measure maximum torque moments and to define the safety limits of the thoracic spine under simulated BAVD maneuver. Simulation of BAVD maneuver was made through the technical axis of rotation. This was defined by the rod located at a distance of 47.10 mm to 50.69 mm from the natural axis of rotation of the thoracic spine located in the central part of the vertebral body. During each experiment the angular range of derotation was determined directly on top of maximal torque due to the digital camera recording of the movement of an arc of the whole BAVD system. It was performed with a continuous motion during the average time 21seconds (17 to 25 seconds) in the angle range between 0° and 70°. A similar range of time is usually needed to perform derotation maneuver during the surgical procedure in-real. (Fig 3A, 3B, 3C and 3D).

**Fig 4:**
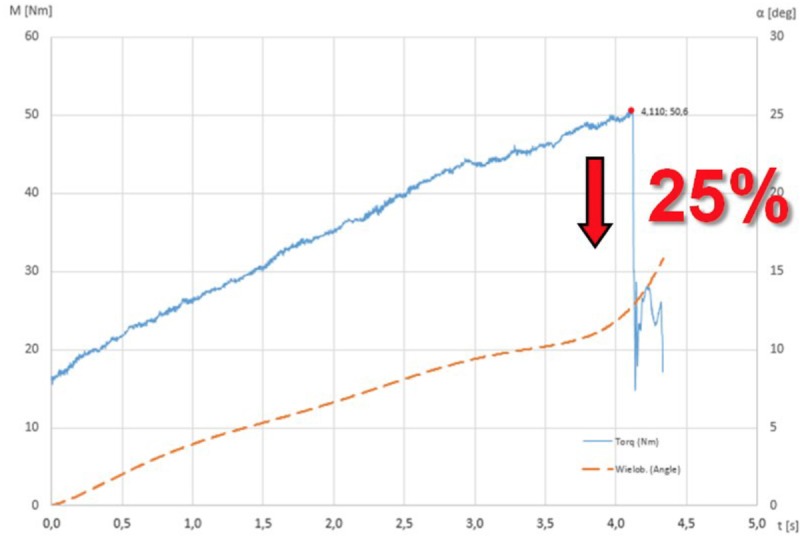
Rapid reduction in torque by more than 25% confirmed the fracture of the vertebral body (torque to failure).

Differences in time of each experiment resulted from the different moment of fracture of the spine. In each case the torsional load was increased incrementally till the catastrophic failure. It was always directly after a quick, drastic drop in the moment rotation curve by more than 25%. It created an inflection points at the maximum moment, what confirmed the fracture of the vertebral body [13,21]. (Fig 4).

**Fig 5:**
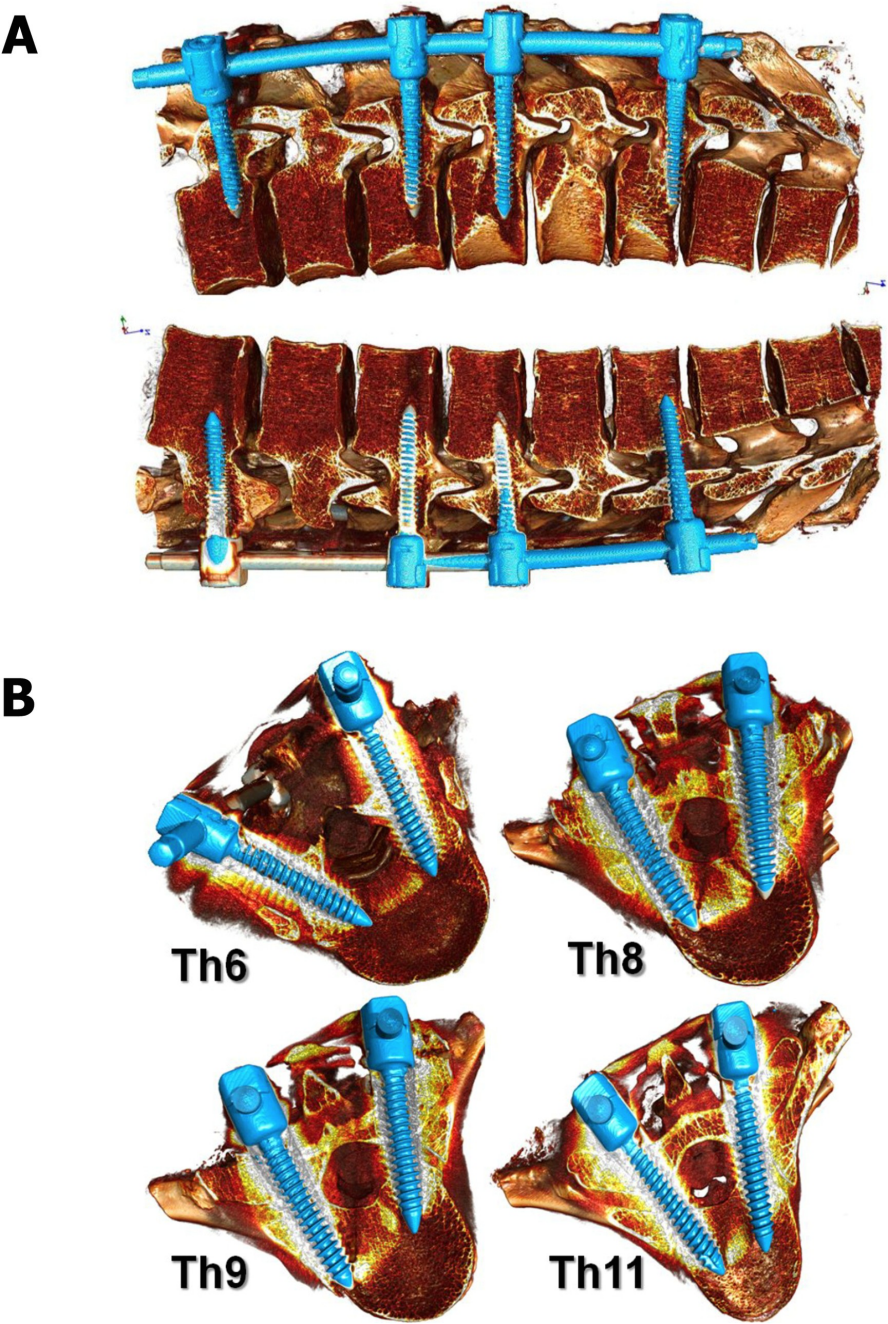
CT evaluation of tested section of the thoracic spine. **A)** Sagittal CT planes after the experiment—pedicle screws position on the left (upper) and right (lower) figure. **B)** Axial CT plane of Th6, 8, 9, 11 vertebrae after the experiment.

For adequate identification of fractures after every experiment examined sections of the spine were resected from the cadaver corpses and subjected to Computed Tomography evaluation (Fig 5A and 5B).

**Fig 6:**
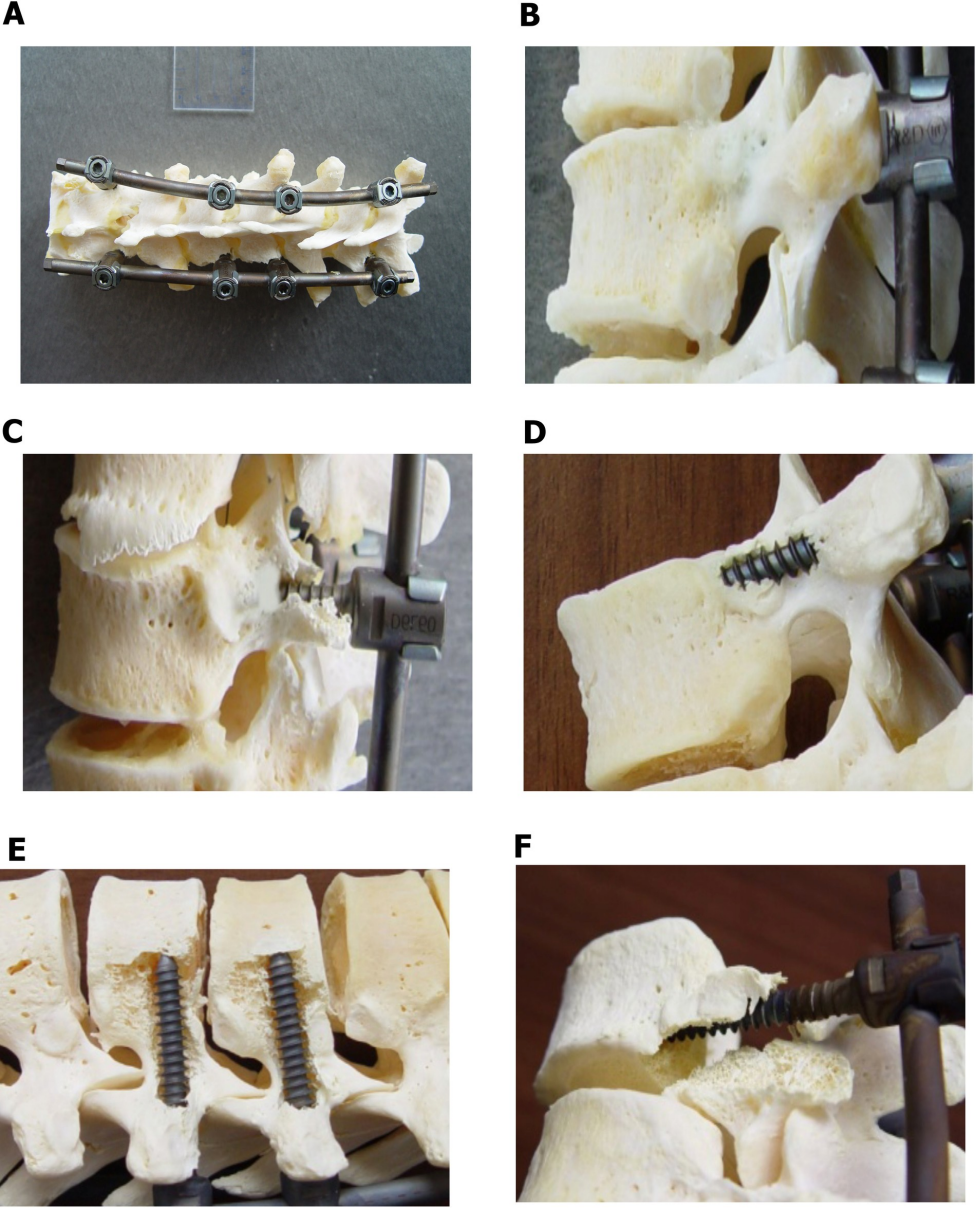
**A)** Thoracic spine section macerated from soft tissues to receive pure bony specimens for the final morphologic assessment of vertebral fractures. Modes of failure: **B)** no failures, **C)** transverse process fracture, **D)** fracture of the lateral wall of pedicle, **E)** breach of the lateral wall, **F)** other failure modes.

After radiological analysis, samples were then macerated from soft tissues to receive pure bony specimens for the final morphologic assessment of vertebral fractures (Fig 6A–6F).

For statistical analysis SPSS 17v software (SPSS Inc.) was used. A paired Student T-test was used for the examination of statistical differences. Factors with p<0,05 were considered statistically significant. R-Spearman test of correlation was used to evaluate the relationship between perioperative variables.

## Results

**Fig 7:**
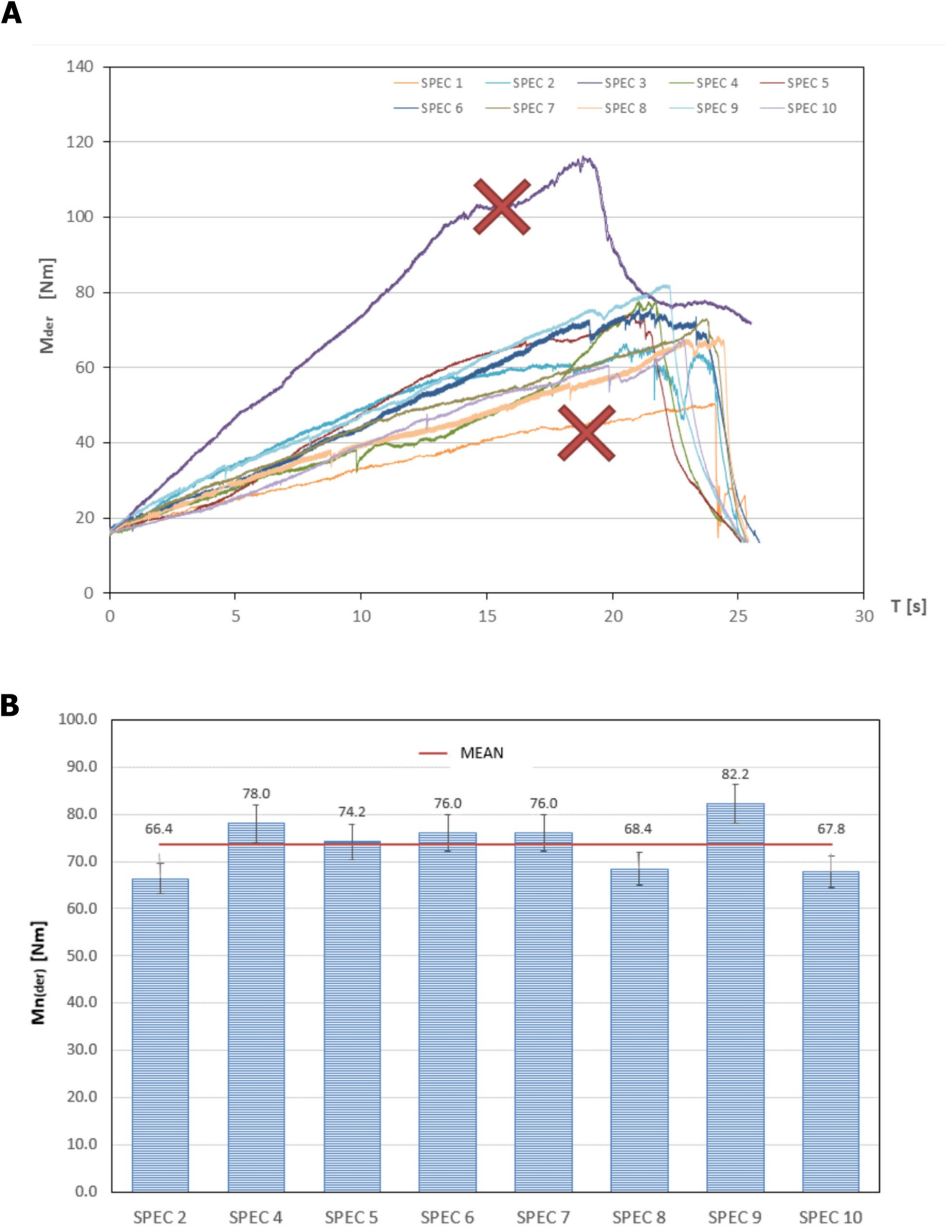
**A)** Graphic representative time–torque to failure loads hysteresis curve from the BAVD simulation of the particular specimens. Extremes were excluded (Spec1 and Spec3) **B)** The average torque to failure during BAVD simulation (M_n(der)_) shown graphically for each sample (Spec2 and Spec4-10) with 5% odds ratio after extremes exclusion: min (Spec1) and max (Spec3).

Ten fresh full-length human cadavers were tested with a medially directed force simulating the forces experienced by the patients during scoliosis surgery and axial plane correction of a typical right thoracic curve. The average torque to failure was summarized in Table 1 and shown graphically (Fig 7A and 7B).

**Table 1:** The summarized average torque to failure during BAVD simulation.

M_n(der)_ [Nm]										M_n(mean)_ [Nm]	SD [Nm]
Spec 1	Spec 2	Spec 3	Spec 4	Spec 5	Spec 6	Spec 7	Spec 8	Spec 9	Spec 10		
50,6	66,4	116,4	78,0	74,2	76,0	73,1	68,4	82,2	67,8	75,3	18,81
	66,4		78,0	74,2	76,0	73,1	66,4	82,2	67,8	73,3	5,49

M_n(mean)_−Mean value of torque to failure [Nm]. M_n(der)_−Torque to failure of particular BAVD simulation [Nm]. First line–Torque values for particular sample testing (Spec1÷10). Second line–Torque values after excluding the extremes: min (Spec1) and max (Spec3)

It ranged from 50,6 to 116,4Nm. After exclusion of two extreme values the average torque to failure during BAVD simulation was M_n*(mean)*_ = 73,3 Nm and standard deviation of 5,49 Nm.

Analysis of digital camera recordings of the average angular range of derotation to failure α _mean_ [°] during BAVD simulation showed to be 44,8° (the angle ranges from 0° to 70°). After exclusion of two extreme values the average angle of BAVD to failure was 44,5° and a standard deviation of 8,16° (Table 2).

**Table 2:** The summarized average angle of derotation to failure during BAVD simulation.

α _max_ [°]										α_mean_[°]	SD [°]
Spec 1	Spec 2	Spec 3	Spec 4	Spec 5	Spec 6	Spec 7	Spec 8	Spec 9	Spec 10		
48,5	61,8	43,3	44,3	32,3	45,5	42,3	42,1	45,8	42,1	44,8	7,33
	61,8		44,3	32,3	45,5	42,3	42,1	45,8	42,1	44,5	8,16

α _mean_−mean value of the angle of derotation to failure during BAVD simulation. α _max_−maximum value of the angle of derotation to failure during BAVD simulation. First line–α _max_ values for particular sample testing (Spec1÷10). Second line–α _max_ values after excluding the extremes: min (Spec1) and max (Spec3)

**Fig 8:**
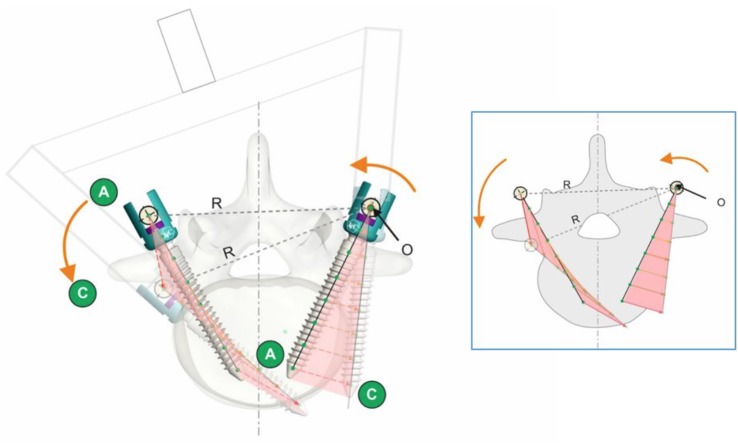
BAVD to failure. After overzealous left-to-right directed force screw displacement mechanism is illustrated (description in text).

After testing an accurate biomechanical analysis of vertebral fractures was performed with CT and morphologic evaluation. All specimens were tested in a quadrangular fashion linked apical pedicle screws and rod on the left (concave) side with a left-to-right (anti-clockwise) directed force simulating the forces during BAVD correction maneuver of a typical right thoracic curve. On the left (concave) side the apical derotation was through the “O—rod axis of rotation”. The maximum strain was at the tip of the pedicle concave screws with the effect of concave side vertebral body wall fracture in all cadavers and minimal changes in the left pedicles. The right (convex) screws rotated through the same axis of rotation as concave side (rod) but were away from it. This created additional torque “R”. The left to right directed forces during BAVD simultaneously pushed the tip of convex screws medially and pulled their tulips laterally with lateral wall breach and transverse process/pedicle wall fracture in all tests (Fig 8). The failure modes were shown in the Fig 6A–6F.

All but one specimen failed bilaterally and specimen no. 9 on the concave side exclusively. The majority of failures were in the apical area. 6 of 10 (60%) specimens on the left side and only 2 of 10 (20%) on the right side had type 3 failure (breach of the lateral wall), (p<0,05). 6 (60%) specimens on the left side and 8 (80%) on the right side had type 1 failure (transverse process fracture), (p<0,05). There was no significant difference between sides in type 2—failure (fracture of the lateral wall of pedicle) and in failures to the end vertebrae (3 of 10 specimens had “0” type bilaterally). There was no spinal canal breach and/or medial wall failure in any specimen (Table 3). The individuals pictured in the Supporting Information video have provided written informed consent (as outlined in PLOS consent form) to appear alongside the article.

**Table 3:** Characteristics of the failure modes after BAVD to failure, based on the analysis of bone specimens after maceration.

*No*	*Left side–(concave)*					*Right side–(convex)*				
	Th6	Th8	Th9	Th11	SCB	Th6	Th8	Th9	Th11	SCB
1	0	2, 3	2	0	–	2	1, 2	1, 2	0	–
2	1, 2	0	1, 2	1, 2	–	2	1, 2, 3	1, 2	1	–
3	0	2, 3	0	1, 2	–	0	1, 2	1, 2	0	–
4	0	2, 3	2, 3	1, 2	–	2	2	0	0	–
5	1, 2, 3	1, 2, 3	2, 3	4	–	0	1, 2	0	4	–
6	1	2, 3	1, 2, 3	1, 2	–	0	1, 2	1	0	–
7	2	1, 2, 3	1, 2, 3	0	–	0	1, 2	1, 2	0	–
8	0	2	2	0	–	2, 3	1, 2	1, 2	1	–
9	0	1, 2	1, 2	0	–	1. 2	0	0	0	–
10	0	1, 2	1, 2	1, 2	–	0	1, 2, 3	1, 2, 3	0	–

SCB—spinal canal breach. Modes of failure were matched with the following numbers: 0—no failures, 1—transverse process fracture, 2—fracture of the lateral wall of pedicle, 3—breach of the lateral wall, 4—other failure modes

## Discussion

The current study was undertaken to evaluate the torsional strength of the instrumented thoracic spine under maximum torque and to define its safety limits under typical derotation maneuver such as BAVD and to better understand the torque required to deliberately fail pedicle screws. For these purposes we designed a special vertebral derotation simulator. For the first time and in contrast to other biomechanical studies of spinal derotation, fresh, young full-length human cadavers were used. The average cadavers age (34,6y) was closer to AIS patients than the age of specimens used in every other study found in the literature [13–18,20,21,25–27]. Unlike in other biomechanical studies [13,21,27], every cadaver was intact and had maintained the integrity of all anatomical structures. Previous studies in the spine biomechanics literature have routinely performed dissections of the part of the spine prior to biomechanical testing [13,21,25–27]. On the other hand, some studies of scoliosis biomechanics based on mathematical model only estimated the failure torques without giving the exact data [22–24,28]. These factors and the method of torque to failure measurement let us to reproduce the actual clinical and biomechanical conditions during scoliosis surgery. In this study after the whole apparatus was assembled the torque was applied through the apical 4 pedicle screws (Th8,9), from the left (concave) to the right (convex) side effectively sharing the load across two apical vertebral bodies. Pedicle screws were always inserted in the same alignment (Th6,8,9,11) leaving Th7 and Th10 non-instrumented for easier apical spinal derotation between the apex and both ends of the curve. Simulation of BAVD maneuver was held through a technical axis defined by the rod positioned on the left (concave) side “O—rod axis of rotation”. The maximum strain was at the tip of the pedicle concave screws on with the effect of concave side vertebral body wall fracture in all cadavers and minimal changes in the left pedicles. The right (convex) screws rotated through the same axis of rotation as concave side (rod) but were away from it. This created additional torque “R”. The left to right directed forces during BAVD simultaneously pushed the tip of convex screws medially and pulled their tulips laterally with lateral wall breach and transverse process/pedicle wall fracture in all tests (Fig 8). It also reproduced the BAVD maneuver after SCRR performed on the left (concave) side positioned rod during AIS surgery [13]. From a biomechanical perspective, pushing and derotating a convex apical pedicle screw provides a more effective and safer derotation than manipulating a concave periapical screw. According to Lee and Suk [1] the anatomic rationale stems from the knowledge that the thicker medial pedicle wall will tolerate 3-times greater axial plane torques applied to pedicle screws in that direction compared with lateral wall. Although from the Parent at al. data, there is some evidence that thoracic pedicle screw, when appropriately placed and contained within the pedicle, will fail at similar torques when rotated medially or laterally (11,5 ± 5,1 vs 12,0 ± 4,9 Nm) [21]. From the safety perspective, thoracic apical derotation should always be performed with the rod positioned on the left (concave) side. In this study with post experimental CT evaluation and maceration of the specimens, we found no spinal canal breach and/or medial wall failure in any specimen. The majority of specimens failed bilaterally in apical Th8 and Th9 area. The failure modes were strongly influenced by the left to right side direction of BAVD maneuver, out of the spinal canal. We found significantly more failures type 3 (breach of the lateral wall) on the left side (3:1), and significantly more type 1 failures (transverse process fracture) on the right side (4:3). There was no difference in type 2 failure modes (fracture of the lateral wall of pedicle) and in failures to the end vertebrae. Cheng et al. in their biomechanical study observed similar failure modes in the thoracic spine after apical derotation simulation. Medially directed screws failed by lateral wall breach and transverse process/pedicle wall fracture (types 1,2,3). Laterally directed forces breached the spinal canal [13]. Parent at al. found that 51% of medially rotated screws failed with canal breach when rod was positioned on the right (convex) side, while laterally rotated screws showed predominantly anterolateral failure (67%) [21]. In the current study, using increasing number of connected screws as a tightened quadrilateral frame, let us to increase the safety of derotation and the torque loads. Cheng at al. shown in their study, that the linking of additional screws had a near linear effect on torque that could be applied for segmental derotation. The average torque to failure in this study was 4 ± 1,4 Nm and 6,1 ± 2,5 Nm for single concave and convex pedicle screw respectively but with quadrangulated construct, such as in our study it was 42,5 ± 16,5Nm of torque to failure [13]. In the current study the average torque to failure during BAVD simulation was 73,3 ± 5,49 Nm and the average angle of BAVD to failure was 44,5° ± 8,16°. Most recently Borkowski et al. in their biomechanical study on 11 fresh-frozen thoracic spines found the torsional load at failure during thoracic spine derotation was 33.3 ± 12.1 Nm and the average derotation angle at T10–T11 was 11.6° ± 5.6°. They used an apparatus simulating DVR attached to pedicle screws at T7–T10 levels. This study was methodologically the closest to our study but again they used only spine specimens dissected of all skin, muscle and fat tissues with the integrity of all vertebrae maintained but the ribs and sternum resected to the posterior 5 cm with the costovertebral joints. Due to this reason the rigidity of the spine in this study might have been underestimated by the lack of the rib-sternal complex [27]. Another limitation common for these biomechanical studies was the use of elderly specimens, with some degree of bone and disc degeneration. Both authors Borkowski and Parent observed some positive correlations between bone density and the insertion torque of the screw, which can be a reflection of bone density and torque to failure magnitude [21,27]. Throughout these studies, thoracic spine failures have been observed at substantially smaller torque magnitudes and angle of derotation maneuver than those in the current study. Further expanding the data from our biomechanical study, a novel apparatus was designed: a custom made dynamometric direct vertebral body “R.Pan” derotator with torque safe limitation based on the current study that is now clinically tested in our department.

## Limitations

Although this is a novel study it has some limitations. First, relatively small sample size– 10 cadavers, which might decrease the power of statistics. It is due to our raw criteria of inclusion of cadavers. There were one female and nine male specimens. That is the inverse of the usual sex distribution in idiopathic scoliosis occurrence. It should be underlined that other biomechanical studies on spinal derotation available in the literature were based on not more than 12 specimens [13,21,27]. Second, the derotation maneuver was performed on non-scoliotic spines without the characteristic pedicle dysplasia and a different stiffness such as in AIS. Therefore, measured forces/safety limits won't be directly used for the treatment of AIS in clinical setting and need to be adequately calibrated for the scoliotic spine first. Explanation of this can be given that for obvious reasons it is nearly impossible to collect intact, young and fresh scoliotic cadavers for the testing. It is simply the limitation that all authors have in this particular space.

## Conclusions

Our study established the safety limits of thoracic spine and efficacy of commonly used intraoperative maneuver (BAVD) for axial plane correction in the treatment of AIS. It provided qualitative and quantitative information essential for the spinal derotation under safe loading limits. Data from this biomechanical study enable to modify the currently used DVR systems for scoliosis correction surgery with fully-controlled derotation maneuver.

## Supporting information

S1 File Video of biomechanical testing. (MP4)
